# Time perspective as a predictor of smoking status: findings from the International Tobacco Control (ITC) Surveys in Scotland, France, Germany, China, and Malaysia

**DOI:** 10.1186/1471-2458-13-346

**Published:** 2013-04-15

**Authors:** Genevieve Sansone, Geoffrey T Fong, Peter A Hall, Romain Guignard, François Beck, Ute Mons, Martina Pötschke-Langer, Hua-Hie Yong, Mary E Thompson, Maizurah Omar, Yuan Jiang

**Affiliations:** 1 Department of Psychology, University of Waterloo, 200 University Avenue West, Waterloo, ON N2L 3G1, Canada; 2 Ontario Institute for Cancer Research, MaRS Centre, South Tower, 101 College Street, Suite 800, Toronto, ON M5G 0A3, Canada; 3 Faculty of Applied Health Sciences, University of Waterloo, 200 University Avenue West, Waterloo, ON N2L 3G1, Canada; 4 Institut National de Prévention et d’Education pour la Santé (INPES), 42 Boulevard de la Liberation, SAINT-DENIS cedex, 93 203, France; 5 Cermes3 - Cesames team (Research Centre Medicine, Sciences, Health, Mental Health, Health Policy), CNRS UMR 8211, Inserm U988, University of Paris Descartes, Sorbonne Paris Cité, EHESS, Paris, France; 6 Unit Cancer Prevention and WHO-Collaborating Centre for Tobacco Control, German Cancer Research Center (DKFZ), Im Neuenheimer Feld 280, Heidelberg, D-69120, Germany; 7 The Cancer Council Victoria, 100 Drummond St., Carlton, VIC, 3053, Australia; 8 Department of Statistics and Actuarial Science, University of Waterloo, 200 University Avenue West, Waterloo, ON, N2L 3G1, Canada; 9 Universiti Sains Malaysia, USM Pulau Pinang, 11800, Malaysia; 10 National Tobacco Control Office, Chinese Center for Disease Control and Prevention, 27 Nanwei Road, Xuan Wu District, Beijing, 100050, P.R. China

**Keywords:** Time perspective, Future orientation, Tobacco, Smoking, Smoking status, International

## Abstract

**Background:**

Prior studies have demonstrated that time perspective—the propensity to consider short-versus long-term consequences of one’s actions—is a potentially important predictor of health-related behaviors, including smoking. However, most prior studies have been conducted within single high-income countries. The aim of this study was to examine whether time perspective was associated with the likelihood of being a smoker or non-smoker across five countries that vary in smoking behavior and strength of tobacco control policies.

**Methods:**

The data were from the International Tobacco Control (ITC) Surveys in five countries with large probability samples of both smokers (*N*=10,341) and non-smokers (*N*=4,955): Scotland, France, Germany, China, and Malaysia. The surveys were conducted between 2005–2008. Survey respondents indicated their smoking status (smoker vs. non-smoker) and time perspective (future oriented vs. not future-oriented) and provided demographic information.

**Results:**

Across all five countries, non-smokers were significantly more likely to be future-oriented (66%) than were smokers (57%), χ^*2*^(1, *N* = 15,244) = 120.64, *p* < .001. This bivariate relationship between time perspective and smoking status held in a multivariate analysis. After controlling for country, age, sex, income, education, and ethnicity (language in France), those who were future-oriented had 36% greater odds of being a non-smoker than a smoker (95% CI: 1.22 to 1.51, *p*<.001).

**Conclusion:**

These findings establish time perspective as an important predictor of smoking status across multiple countries and suggest the potential value of incorporating material to enhance future orientation in smoking cessation interventions.

## Background

In recent explanatory models of health-risk and health-promoting behaviors, considerations of time frame have played a prominent role, in part because of the temporal asymmetry of costs and benefits involved [1]. Such temporal considerations can help to explain why so many people avoid health protective behaviors even though they might value their eventual outcomes, and engage in health risk behaviors, such as smoking, that confer long-term disadvantages.

Temporal asymmetries figure prominently in smoking behavior, given that the act of smoking can provide a range of immediate rewards to the smoker, including stress reduction, improved concentration, and feelings of social connectedness, with only minor immediate costs [2]. The overall balance of consequences shifts in the opposite direction over time, given that smoking is the leading cause of chronic disease and mortality in the world today, and prospective studies have found up to threefold increased mortality risk for smokers compared to non-smokers [3-5]. Despite these well-known risks, individuals may continue to smoke if the perceived immediate benefits of smoking outweigh the future health consequences. It follows then that greater consideration of the future costs over the immediate benefits of health-damaging behaviors such as smoking should facilitate behavior that is consistent with one’s long-term health interests.

The general tendency to consider the future consequences of one’s current behavior has been described as an individual difference variable called *time perspective*[1,6-8]. Various definitions and measurements of this concept have been applied by researchers, including “personal time horizon” [9], “consideration of future consequences” [10], and “future orientation or future-orientedness” [6,11]. These diverse conceptualizations share a common emphasis on the way individuals consider temporal factors in order to explain behaviors that might have implications for health status and longevity [1,12].

Time perspective has been differentiated from related but separate individual difference variables such as impulsivity and sensation seeking on both a conceptual level and in terms of the hypothesized neural underpinnings of the constructs [13]. Impulsivity is generally thought to reflect the operation of lower brain reward centres of older evolutionary origin, and reflects a tendency to act without thinking. Time perspective, on the other hand, is associated with the higher cortical centres associated with abstract reasoning, and reflects a tendency to deliberate over short versus long term contingencies (rather than a lack of deliberation overall) [14,15]. While both constructs have been described as relatively stable individual difference variables, evidence suggests that time perspective can be modified through targeted interventions, whereas targeting interventions based on impulsivity and sensation seeking involves identifying high-risk individuals and then tailoring interventions based on their existing level of the trait [16,17].

The relationship between time perspective and health-related behavior has been documented in several health domains. In general, research supports the notion that individuals with a stronger future time perspective are more likely to engage in protective health behaviours and less li[kely to engage in risky health behaviours including substance use [1,6,7,17-20]. An experimental study by Hall and Fong found that increasing future-orientation can lead to increases in physical activity, supporting the notion that there is a causal relationship between time perspective and health protective behavior [17]. In a more recent study, Hall and Fong also demonstrated a positive association between future-oriented time perspective and uptake of weight management behaviors (reduction of fatty food consumption and increase in regular physical activity), and this association was mediated by intention strength, suggesting that future orientation may generate stronger motivation to perform positive health behaviors such as weight management [20].

Only a few studies have specifically examined the role of time perspective in the domain of tobacco use [12,13,21,22], but the evidence thus far indicates that time perspective can predict both smoking initiation among youth and cessation behavior among adult smokers. For instance, in a sample of high school students who completed the North American Student Smoking Survey (NASSS), scores on a time perspective scale were shown to predict smoking status as well as likelihood of initiation among those who had never smoked [8]. These findings are supported by a more recent study using a small sample of U.S. adults, in which future time perspective, as measured by two different scales, was associated with decreased risk of being a current smoker [21].

The relation between time perspective and quit behavior has been examined in a cohort study of smoking cessation among older English adults. Using a measure of financial planning as a proxy for time perspective, Adams found that those who reported more long-term periods for financial planning were less likely to be smokers, and among the smokers, those with a more future-oriented time perspective were more likely to quit [12]. A recent study of smokers in four English speaking countries (U.S., Canada, U.K. and Australia) found time perspective to be a significant predictor of quit attempts, and this relation was mediated by quit intentions [13]. In addition, Kovac and Rise have found that scores on a time perspective measure moderated the relation between intentions to quit and actual quitting behavior, suggesting that a future time perspective enhances smokers’ ability to follow through on their motivation to quit [22]. Therefore, the existing research suggests that future time perspective is negatively related to smoking behavior and positively associated with quitting among those who smoke.

In summary, from the modest number of studies that have examined the association between time perspective and smoking behavior, there is growing support for the notion that future time perspective is an important construct in understanding factors that make people more or less likely to smoke or to stop smoking. However, some of these studies were small scale samples, and none have examined the association between time perspective and smoking status across geographic regions that vary in language, cultural milieu, and ethnic composition. For this reason, we do not currently have precise estimates regarding the strength of association between time perspective and smoking behavior, nor do we know how well this association—if present—holds across different populations.

The goal of the present study was to test whether individual differences in time perspective predict smoking status (smoker vs. non-smoker) using large representative probability samples in multiple countries, with the a priori hypothesis that those with a stronger future time perspective would be more likely to be non-smokers. A secondary aim was to examine whether the relation between future time perspective and smoking status would differ across cultures. As most research on time perspective has been conducted only in high-income countries, it is important to examine whether the relation between time perspective and smoking status varies across a range of both high-income and lower-income countries, each with unique tobacco control situations and levels of tobacco use.

## Methods

### Data source

The data were obtained from the longitudinal cohort surveys being conducted by the International Tobacco Control (ITC) Policy Evaluation Project. The ITC Project is designed to evaluate the effects of national level tobacco control policies in over 20 countries and to examine the causal mechanisms responsible for policy impact [23]. All ITC Surveys follow the same conceptual framework and methodology, which makes it possible to compare smoking behaviors and attitudes at the population level across countries. The survey and research protocol was reviewed and cleared for ethics by the Office of Research Ethics at the University of Waterloo, and by the relevant local institutional review boards for three of the countries: the Ethics Commission of the Medical Faculty Heidelberg, University of Heidelberg (Germany), the Research and Ethics Committee of the School of Medical Sciences, Universiti Sains Malaysia (Malaysia), and the Ethical Review Committee of the Chinese Center for Disease Control and Prevention and the Institutional Research Review Committee at Cancer Council Victoria (China).

### Participants

The analyses presented here are from the first two cohorts of ITC Survey respondents in the five countries available at the time of the study where large probability samples of both smokers and non-smokers were surveyed: Scotland, France, Germany, China, and Malaysia. Data were from the first survey wave in each country except for China and Scotland, which were missing the measure of time perspective at Wave 1. Respondents in all countries were adults aged 18 years and older. Respondents were defined as smokers if they reported having smoked more than 100 cigarettes in their lifetime, and currently smoked at least weekly (Malaysia and China) or monthly (Scotland, France, and Germany). The sample sizes and periods of data collection for each country are shown in Table 1.

**Table 1:** Samples by country

	**Scotland**	**France**	**Germany**	**China**	**Malaysia**	**Total**
Number of smokers	461	1,735	1,515	4,626	2,004	10,341
Number of non-smokers	378	525	1,059	1,438	1,555	4,955
Total *N*	839	2,260	2,574	6,064	3,559	15,296
Survey wave	2	1	1	2	1	
Dates of surveys	March-April 2007	Dec 2006-Feb 2007	July-Nov 2007	Oct 2007-Feb 2008	Jan-March 2005	

### Sampling design and procedures

In three of the countries (Scotland, France, and Germany), probability samples of respondents were recruited using random digit dialing methods. Smokers were oversampled in order to fill the quota for smoking respondents, which differed in each country. The sampling design was stratified by geographic region and community size in Scotland and Germany. In Malaysia and China, respondents were selected using a stratified multistage cluster sampling design, with inclusion probability proportional to size at each stage before the last. In China, the sampling frames were constructed to be representative of each of 6 cities: Beijing, Shanghai, Guangzhou, Changsha, Yinchuan, and Shenyang; in Malaysia, the sampling frames were the urban and rural areas of one state in each of the 6 geographic zones of the country. All selected respondents in each country then completed the main questionnaire through either computer-assisted telephone interviewing (CATI; in Scotland, France and Germany) or face-to-face interviewing (in China and Malaysia). Additional information about the design and survey methodologies in each country is available at the ITC Project website [24].

### Measures

#### Demographics

The following demographic measures were included in the analyses: sex, age group, ethnicity (language), education, and income. Age was divided into four categories: 18–24 years, 25–39 years, 40–54 years, and 55 or older. Ethnicity was measured according to census categories in each country and was coded here as majority versus otherwise. In France, however, respondents were categorized by language (French-speaking only vs. other languages spoken in the household), consistent with the lack of ethnicity questions on the French census and in other French surveys. Education level was also measured using standard categories in each country. To make these categories comparable across countries, we divided respondents into three groups: low, moderate, or high education. Income levels were similarly matched across countries and categorized as low, moderate, high, or no answer.

#### Variables of interest

The primary predictor variable was time perspective. This construct is assessed in the ITC Surveys using a single item that had the highest item-total correlation in the Fong and Hall Time Perspective Questionnaire (TPQ), which has demonstrated adequate reliability and validity and predicts a variety of health related behaviors and the single item used here has previously predicted smoking related quit activity [8,13]. Respondents indicated on a five-point scale the extent to which they agreed with the following statement: “You spend a lot of time thinking about how what you do today will affect your life in the future.” The question was translated by professional bilingual translators for surveys conducted in languages other than English. From a conceptual standpoint, and to be consistent with previous research, a binary variable was created so that responses of “Strongly Agree” and “Agree” were coded as “future-oriented” and responses of “Strongly Disagree,” “Disagree,” and “Neither Agree nor Disagree” were coded as “not future-oriented” [7,25].

The primary dependent variable was respondents’ smoking status at the time of the survey, which was coded as either current Smoker (daily and non-daily) or current Non-Smoker. The Non-Smoker category may include former smokers, and for two of the countries (Scotland and France), it included a small number of quitters (respondents who had quit smoking since the previous wave of the survey, N=263).

### Data analyses

SPSS Version 19 was used for all statistical analyses. Analyses were conducted using rescaled cross-sectional weights for smokers and non-smokers (see Scott and Wild, 2001, for a justification of using these weights within a complex sampling design) [26]. After examining the bivariate relation between time perspective and smoking status, a multivariate analysis with all countries pooled in one dataset was performed using a logistic regression model to predict the likelihood of being a non-smoker from the predictor variables. Separate multiple logistic regressions were also performed within the individual country samples. Finally, including an interaction term between country and time perspective in a multiple logistic regression using pooled data provided a test of whether the relation between time perspective and smoking status differed across the five countries.

## Results

### Characteristics of the sample

#### Sociodemographics

Table 2 presents the sociodemographic characteristics of the respondents in each country by smoking status. The majority of the overall sample (65.1%) was composed of males, largely due to the greater proportion of male smokers in each country. This was especially noticeable in the China and Malaysia samples, reflecting the differences in smoking prevalence between the sexes in these two countries; for example, 52.9% of males in the Chinese population smoke compared to only 2.4% of women [27].

**Table 2:** Sample characteristics by country

**Variables**	**Country**											
	**All countries**		**Scotland**		**France**		**Germany**		**China**		**Malaysia**	
	**S**	**NS**	**S**	**NS**	**S**	**NS**	**S**	**NS**	**S**	**NS**	**S**	**NS**
Sex												
% Male	77.9	38.6	40.3	36.8	48.2	34.5	47.8	41.9	94.9	47.1	95.7	30.2
Age group												
18-24	8.0	8.0	6.5	4.0	13.4	8.0	14.9	7.6	1.0	3.4	15.0	13.4
25-39	24.7	24.7	27.1	21.7	37.2	24.4	25.1	22.4	16.1	18.2	33.1	33.1
40-54	42.1	34.6	38.6	31.2	36.6	32.4	40.2	31.4	49.1	39.6	32.6	33.8
55+	25.2	32.7	27.8	43.1	12.8	35.2	19.7	38.6	33.9	38.9	19.3	19.7
Ethnicity												
Majority	88.5	83.7	97.8	95.5	87.0	87.6	96.3	97.5	94.8	93.8	66.9	60.7
Income												
Low	25.3	24.3	54.7	42.3	28.9	26.3	26.0	22.0	16.5	14.2	35.3	30.2
Moderate	41.9	38.4	25.6	22.0	44.2	45.0	38.6	34.4	45.3	49.9	38.3	32.2
High	25.2	24.6	13.0	25.7	23.8	23.4	20.0	24.6	31.5	30.1	18.5	19.6
No answer	7.6	12.8	6.7	10.1	3.1	5.3	15.4	19.1	6.7	5.8	7.9	18.1
Education level												
Low	20.8	21.0	55.4	49.5	44.9	50.3	22.2	18.1	11.8	13.2	11.5	13.3
Moderate	57.4	52.2	30.9	26.7	35.3	30.3	37.8	33.4	66.8	60.9	76.0	70.6
High	21.7	26.8	13.0	22.8	19.8	19.4	40.0	48.5	21.4	25.8	12.4	16.2
No answer	0.0	0.1	0.7	1.1	0.1	0.0	0.0	0.0	0.0	0.0	0.0	0.0

Note: all estimates are unweighted.

*S*, Smokers; *NS*, Non-smokers.

#### Smoking status

The majority of respondents in the entire dataset (67.7%) were smokers, which reflects the oversampling of smokers in each country. The samples from France and China had the greatest proportion of smokers (76.8% and 76.6% respectively), and Scotland and Malaysia had the lowest (54.7% and 56.3%). These differences reflect the different quotas for smokers and non-smokers within each country.

#### Time perspective

Analyses of the prevalence of future time perspective revealed that overall, more than half of respondents (59.6%) were categorized as being future-oriented, that is, they agreed or strongly agreed with the statement: “You spend a lot of time thinking about how what you do today will affect your life in the future”. However, there was a large range in the prevalence of future orientation across countries. Malaysia was the country with the highest proportion of respondents with a future orientation (84.7% of the sample); China had the lowest proportion of respondents with a future orientation (41.2%).

### Bivariate association between time perspective and smoking status

**Figure 1:**
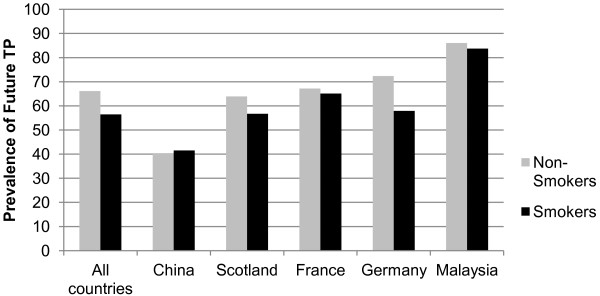
**Prevalence of future time perspective by smoking status.** ***p<.001. All estimates are weighted.

As expected, there was a significant association between time perspective and smoking status across the whole sample, as seen in Figure 1. Overall, the prevalence of future orientation was significantly higher among non-smokers (66%) than it was among smokers (57%). The greatest difference in future orientation between non-smokers and smokers was found in Germany (72.4% vs 57.9%).

### Multivariate analysis

Next, the ability of the time perspective measure to predict the likelihood of being a non-smoker beyond sociodemographic variables was examined in a multivariate logistic regression model (See Table 3). After controlling for country, age, sex, income, education, and ethnicity (language in France), future-oriented respondents had 36% greater odds of being a non-smoker than respondents who were not future-oriented (95% CI: 1.22 to 1.51, *p*<.001).

**Table 3:** Logistic regression analysis of time perspective across all countries

**Predictors**	**Non-smoker OR (95% CI)**
Demographics	
Age (ref=55+yrs)	
18-24 yrs	0.469 (0.375-0.588)***
25-39 yrs	0.400 (0.346-0.461)***
40-54 yrs	0.504 (0.445-0.571)***
Sex (ref=female)	
Male	0.155 (0.139-0.173)***
Ethnicity (ref=otherwise)	
Majority	0.776 (0.658-0.916)**
Income (ref=low)	
Moderate	1.278 (1.118-1.460)***
High	1.398 (1.201-1.626)***
No answer	1.671 (1.377-2.028)***
Education (ref=low)	
Moderate	1.147 (0.990-1.328)
High	1.797 (1.526-2.116)***
No answer	1.992 (0.162-24.483)
Time perspective (ref=present-oriented)	
Future-oriented	1.357 (1.220-1.508)***

**p<.01, ***p<.001.

Note: the country variable was also controlled for in the analysis.

Further exploration of whether this relation between time perspective and smoking status varied depending on country was accomplished by including a single omnibus interaction term (time perspective x country) in the logistic regression analysis. This overall time perspective x country interaction term was significant (Wald statistic = 47.10, *p*<.001), indicating that the strength of the relationship between time perspective and smoking status did vary across the countries included in this study.

An additional analysis was conducted, stratifying by country, in order to estimate the predictive power of time perspective within each country. Findings indicated that time perspective was a significant predictor of smoking status in every country except China (OR=1.06, n.s.; see Table 4); specifically, in all countries but China, future-oriented individuals had significantly greater odds of being a non-smoker than their present-oriented counterparts. Post-hoc comparisons showed that the relationship between time perspective and smoking status was significantly stronger in Scotland (*p*<.001), Germany (*p*<.001), and Malaysia (*p*<.001) than it was in France. No other significant differences between countries were found.

**Table 4:** Logistic regression analysis in each country

**Country**	***N***	**Non-smoker adjusted OR (95% CI)**
Scotland	825	1.44 (1.00-2.05)*
France	2,254	1.28 (1.00-1.64)*
Germany	2,538	1.89 (1.55-2.30)***
China	5,598	1.06 (0.86-1.29)
Malaysia	3,087	2.42 (1.59-3.68)***
All countries	14,358	1.36 (1.22-1.51)***

*p < .05; ***p<.001.

Note. Adjusted odds ratios are from model adjusted for respondents’ gender, age, income, ethnicity, and education.

## Discussion

The main objective of this study was to examine whether individual differences in time perspective can distinguish between smokers and non-smokers cross-nationally. As predicted, our findings indicated that time perspective was indeed associated with smoking status, such that individuals who were more future-oriented were more likely to be non-smokers than smokers. Of the countries surveyed, the only exception to this trend was China; in Scotland, France, Germany and Malaysia, future-orientedness was associated with significantly lower odds of being a current smoker than present-orientedness.

One important strength of the current study is the use of large probability samples in five countries to estimate the strength of association between time perspective and smoking status. In contrast, most prior studies examining time perspective and health risk behaviors have been relatively small in size and unknown in representativeness. Furthermore, the multi-country nature of the sample allowed for comparison of the strength of association across and within different countries that vary in level of knowledge about smoking and potency of tobacco control legislation.

The relation between time perspective and smoking status was relatively uniform across the countries included in this study. Despite differences in sampling methods, the association was found in four of the five countries surveyed, although the strength of the relationship varied among these countries. As mentioned, future-oriented respondents in Malaysia had the highest odds of being a non-smoker. The weakest (non-significant) association between time perspective and smoking status was found in China, where the prevalence of future-orientation was also lowest.

One potential explanation for the non-significant relation between time perspective and smoking status in China relates to the extent to which the harms of smoking are salient among the Chinese people. China has only recently begun to take efforts towards reducing tobacco use, and ITC Project research has demonstrated that awareness in China of specific health risks of smoking is still very low compared to other countries [28,29]. It may be the case then that in countries such as China where there is a lack of awareness of the future consequences of tobacco use, the role of time perspective is diminished. In other words, the effects of having a future time perspective on smoking behavior can only operate when the awareness exists of the trade-off between the long-term over the short-term consequences of smoking.

### Contribution and implications

The present findings are consistent with previous research in the health behavior literature that has demonstrated a relation between time perspective or future orientation and health-promoting behavior [6,7,17-20]. Our findings also contribute to the growing body of literature on the relationship between time perspective and smoking behavior. However, prior studies on time perspective and smoking have been primarily conducted within single (high income) countries, and so the extent to which the association holds across high- and lower-income countries has not been known, nor has the degree of variability between countries. The results of the present study confirm in a much broader and international sample the fact that there is indeed a strong relation between time perspective and whether or not an individual will be a smoker. This is true across countries that are culturally and economically diverse. From this perspective, the current findings augment our understanding of the magnitude and generality of the relationship between time perspective and smoking behavior and thus its importance in understanding the factors associated with smoking in an international context.

The findings from this study may also have important implications for guiding smoking cessation strategies and for designing more effective population-level tobacco control campaigns. In the domain of tobacco, health interventions may be more successful to the extent that they are able to alter the temporal balance of the costs and benefits of smoking, such as by making the long-term costs more salient than the immediate rewards that smoking provides [17]. Indeed, redressing the temporal imbalance may be one of the reasons why graphic health warnings on cigarette packages, which make future health outcomes such as disease and death very salient, are more effective than text-only warnings in informing smokers about the harms of their behaviour and motivating them to quit [30]. Similarly, smoking bans may be effective to the extent that they make the act of smoking more inconvenient and less rewarding in the short-term. Finally, increasing the price of cigarettes is the most straightforward example of how immediate costs—in this case financial costs—can lead to behavioural changes, compared to the situation prior to a price increase, when the only costs that are brought to bear on the decision of whether to quit smoking are those that may lie far into the future.

Our findings also suggest that it may be possible to reduce the prevalence of smoking and its devastating consequences on health through an intervention specifically designed to increase long-term thinking about the consequences of one’s behavior. Therefore, smoking cessation interventions that include components designed to help people become more future-oriented may be particularly effective in helping smokers to quit.

### Limitations

The varying sampling designs that were used in the ITC countries chosen for these analyses represent one limitation of the present study, as only three of the five countries had fully representative samples at the national level (Scotland, France, and Germany), although the other two samples were fully representative of the sub-national populations from which the samples were drawn (e.g., in China, cities totalling over 60 million people). An additional limitation is the cross-sectional analyses rather than a prospective study design; therefore, it is not clear whether or not individual differences in time perspective actually preceded smoking status. It would also be important to examine whether differences in time perspective are found among those who have quit smoking compared to current smokers and non-smokers, which we were unable to do in the present study because we did not have data from quitters in all countries. To help to establish a causal relation between future time perspective and smoking-related behaviour, future studies should additionally examine whether smokers with a stronger future orientation are more likely to attempt to quit and are more successful in their quit attempts over time.

## Conclusions

This study found that individual differences in time perspective – the tendency to consider short-term versus future consequences of one’s actions – predicts smoking status at the cross-national level. Analyses across five high- and lower-income countries found that the prevalence of future time perspective was significantly higher among non-smokers than among smokers, although the strength of the relationship varied among these countries and was not significant in China. These findings further establish time perspective as an important predictor of smoking status and suggest the potential value of smoking cessation interventions to enhance future time perspective.

## Competing interests

The authors declare that they have no competing interests.

## Authors’ contributions

GS was responsible for data analysis and preparing the manuscript. GS, GTF, PAH made major contributions to the research conception and interpretation of data analysis, as well as substantial contributions to the drafting of the manuscript. All authors read and provided comments during the manuscript preparation and approved the final manuscript.

## Pre-publication history

The pre-publication history for this paper can be accessed here:

http://www.biomedcentral.com/1471-2458/13/346/prepub
